# 5-(3-Fluoro­phen­yl)-1-phenyl­pyrazolidin-3-one

**DOI:** 10.1107/S1600536808026706

**Published:** 2008-09-06

**Authors:** Yuan-Yuan Liu, Hong Shi, Qing-Yan Chu, Hong-Jun Zhu

**Affiliations:** a Department of Applied Chemistry, College of Science, Nanjing University of Technology, Nanjing 210009, People’s Republic of China

## Abstract

In the mol­ecule of the title compound, C_15_H_13_FN_2_O, the phenyl and fluorophenyl rings are oriented at a dihedral angle of 77.92 (3)°. The pyrazolidine ring adopts an envelope conformation. An intra­molecular C—H⋯N hydrogen bond results in the formation of a five-membered ring adopting an envelope conformation. In the crystal structure, inter­molecular N—H⋯O and C—H⋯O hydrogen bonds link the mol­ecules. There are C—H⋯π contacts between between aromatic H atoms and the phenyl and fluorophenyl rings. A π–π contact between phenyl rings [centroid–centroid distance = 3.926 (1) Å] is also observed.

## Related literature

For general background, see: Chiara & Garcia (2005 ▶). For related literature, see: Jia *et al.* (2008 ▶). For bond-length data, see: Allen *et al.* (1987 ▶).
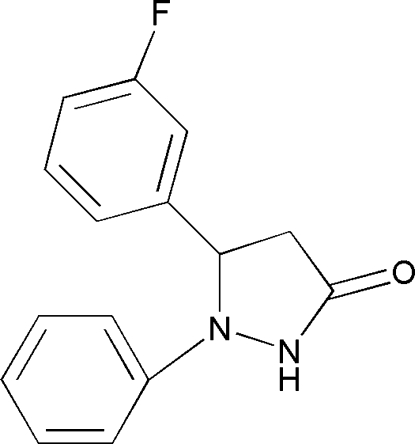


## Experimental

### 

#### Crystal data

C_15_H_13_FN_2_O
*M* *_r_* = 256.27
Monoclinic, 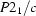

*a* = 10.265 (2) Å
*b* = 7.3130 (15) Å
*c* = 17.822 (4) Å
β = 92.39 (3)°
*V* = 1336.7 (5) Å^3^
*Z* = 4
Mo *K*α radiation
μ = 0.09 mm^−1^
*T* = 294 (2) K
0.30 × 0.20 × 0.10 mm

#### Data collection

Enraf–Nonius CAD-4 diffractometer
Absorption correction: ψ scan (North *et al.*, 1968 ▶) *T*_min_ = 0.973, *T*_max_ = 0.991
2393 measured reflections
2393 independent reflections
1231 reflections with *I* > 2σ(*I*)
3 standard reflections frequency: 120 min intensity decay: none

#### Refinement

*R*[*F* ^2^ > 2σ(*F* ^2^)] = 0.081
*wR*(*F* ^2^) = 0.232
*S* = 1.07
2393 reflections
166 parameters
H-atom parameters constrained
Δρ_max_ = 0.34 e Å^−3^
Δρ_min_ = −0.75 e Å^−3^

### 

Data collection: *CAD-4 Software* (Enraf–Nonius, 1985 ▶); cell refinement: *CAD-4 Software*; data reduction: *XCAD4* (Harms & Wocadlo, 1995 ▶); program(s) used to solve structure: *SHELXS97* (Sheldrick, 2008 ▶); program(s) used to refine structure: *SHELXL97* (Sheldrick, 2008 ▶); molecular graphics: *SHELXTL* (Sheldrick, 2008 ▶); software used to prepare material for publication: *SHELXTL*.

## Supplementary Material

Crystal structure: contains datablocks I, lyy. DOI: 10.1107/S1600536808026706/hk2512sup1.cif

Structure factors: contains datablocks I. DOI: 10.1107/S1600536808026706/hk2512Isup2.hkl

Additional supplementary materials:  crystallographic information; 3D view; checkCIF report

## Figures and Tables

**Table 1:** Hydrogen-bond geometry (Å, °)

*D*—H⋯*A*	*D*—H	H⋯*A*	*D*⋯*A*	*D*—H⋯*A*
N2—H2*A*⋯O^i^	0.86	1.92	2.777 (4)	172
C4—H4*A*⋯N1	0.93	2.48	2.836 (6)	103
C8—H8*A*⋯O^ii^	0.97	2.59	3.422 (5)	143
C6—H6*A*⋯*Cg*3^iii^	0.93	2.96	3.866 (3)	165
C12—H12*A*⋯*Cg*2^iv^	0.93	3.05	3.751 (3)	134
C14—H14*A*⋯*Cg*3^v^	0.93	2.80	3.654 (3)	153

Symmetry codes: (i) 
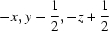
; (ii) 
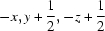
; (iii) 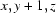; (iv) 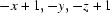; (v) 
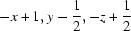
. *Cg*2 is the centroid of the C1–C6 ring and *Cg*3 is the centroid of the C10–C15 ring.

**Fig. 1.**
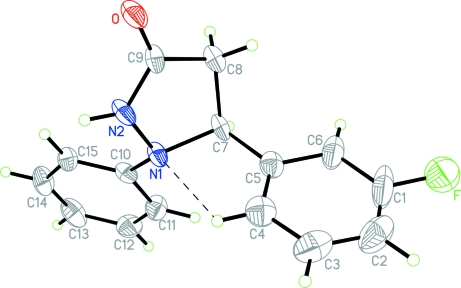
The molecular structure of the title molecule, with the atom-numbering scheme. Displacement ellipsoids are drawn at the 50% probability level. Hydrogen bond is shown as dashed line.

**Fig. 2.**
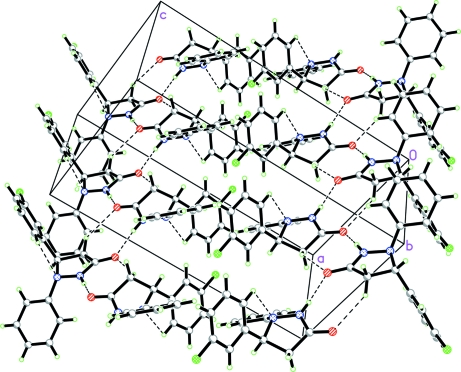
A partial packing diagram of the title compound. Hydrogen bonds are shown as dashed lines.

## supplementary crystallographic information

### Comment

Pyrazolidin-3-one and its derivatives used as medicines and herbicides (Chiara & Garcia, 2005) have been developed most quickly, such as anodyne and antipyretic. We report herein the crystal structure of the title compound.

In the molecule of the title compound (Fig. 1), the bond lengths (Allen *et al.*,  1987) and angles are within normal ranges. Rings A (C1-C6) and C (C10-C15) are, of course, planar, and they are oriented at a dihedral angle of A/C = 77.92 (3)°. Ring B (N1/N2/C7-C9) adopts envelope conformation, with C8 atom displaced by 0.277 (3) Å from the plane of the other ring atoms. The intra- molecular C-H···N hydrogen bond (Table 1) results in the formation of a five-membered ring D (N1/C4/C5/C7/H4A) adopting envelope conformation, with N1 atom displaced by -0.326 (3) Å from the plane of the other ring atoms.

In the crystal structure, intermolecular N-H···O and C-H···O hydrogen bonds link the molecules (Fig. 2), in which they may be effective in the stabilization of the structure. The C—H···π contacts (Table 1) between the phenyl rings and the aromatic H atoms and a π—π contact between phenyl rings Cg2···Cg2^i^ [symmetry code: (i) -x, 1 - y, 1 - z, where Cg2 is centroid of the ring A (C1-C6)] further stabilize the structure, with centroid-centroid distance of 3.926 (1) Å.

### Experimental

The title compound was prepared according to the literature method (Jia *et al.*,  2008). The crystals were obtained by dissolving the title compound (1.5 g) in ethyl acetate (25 ml) and evaporating the solvent slowly at room temperature for about 10 d.

### Refinement

H atoms were positioned geometrically, with N-H = 0.86 Å (for NH) and C-H = 0.93, 0.98 and 0.97 Å for aromatic, methine and methylene H, respectively, and constrained to ride on their parent atoms with U_iso_(H) = 1.2U_eq_(C,N).

### Crystal data

**Table:**

C_15_H_13_FN_2_O	*F*(000) = 536
*M**_r_* = 256.27	*D*_x_ = 1.273 Mg m^−^^3^
Monoclinic, *P*2_1_/*c*	Mo *K*α radiation, λ = 0.71073 Å
Hall symbol: -P 2ybc	Cell parameters from 25 reflections
*a* = 10.265 (2) Å	θ = 9–12°
*b* = 7.3130 (15) Å	µ = 0.09 mm^−^^1^
*c* = 17.822 (4) Å	*T* = 294 K
β = 92.39 (3)°	Needle, colorless
*V* = 1336.7 (5) Å^3^	0.30 × 0.20 × 0.10 mm
*Z* = 4	

### Data collection

**Table:**

Enraf–Nonius CAD-4 diffractometer	1231 reflections with *I* > 2σ(*I*)
Radiation source: fine-focus sealed tube	*R*_int_ = 0.000
graphite	θ_max_ = 25.2°, θ_min_ = 2.0°
ω/2θ scans	*h* = −12→12
Absorption correction: ψ scan (North *et al.*, 1968)	*k* = 0→8
*T*_min_ = 0.973, *T*_max_ = 0.991	*l* = 0→21
2393 measured reflections	3 standard reflections every 120 min
2393 independent reflections	intensity decay: none

### Refinement

**Table:**

Refinement on *F*^2^	Secondary atom site location: difference Fourier map
Least-squares matrix: full	Hydrogen site location: inferred from neighbouring sites
*R*[*F*^2^ > 2σ(*F*^2^)] = 0.081	H-atom parameters constrained
*wR*(*F*^2^) = 0.232	*w* = 1/[σ^2^(*F*_o_^2^) + (0.1065*P*)^2^ + 0.1962*P*] where *P* = (*F*_o_^2^ + 2*F*_c_^2^)/3
*S* = 1.07	(Δ/σ)_max_ < 0.001
2393 reflections	Δρ_max_ = 0.34 e Å^−^^3^
166 parameters	Δρ_min_ = −0.75 e Å^−^^3^
Primary atom site location: structure-invariant direct methods	

### Special details

**Table:**

Geometry. All e.s.d.'s (except the e.s.d. in the dihedral angle between two l.s. planes) are estimated using the full covariance matrix. The cell e.s.d.'s are taken into account individually in the estimation of e.s.d.'s in distances, angles and torsion angles; correlations between e.s.d.'s in cell parameters are only used when they are defined by crystal symmetry. An approximate (isotropic) treatment of cell e.s.d.'s is used for estimating e.s.d.'s involving l.s. planes.
Refinement. Refinement of F^2^ against ALL reflections. The weighted R-factor wR and goodness of fit S are based on F^2^, conventional R-factors R are based on F, with F set to zero for negative F^2^. The threshold expression of F^2^ > 2sigma(F^2^) is used only for calculating R-factors(gt) etc. and is not relevant to the choice of reflections for refinement. R-factors based on F^2^ are statistically about twice as large as those based on F, and R- factors based on ALL data will be even larger.

### Fractional atomic coordinates and isotropic or equivalent isotropic displacement parameters (Å^2^)

**Table:**

	*x*	*y*	*z*	*U*_iso_*/*U*_eq_	
O	0.0023 (3)	0.2565 (4)	0.22989 (17)	0.0567 (8)	
F	0.2688 (3)	0.7468 (5)	0.56248 (19)	0.098	
N1	0.2364 (3)	0.0858 (4)	0.35618 (19)	0.0407 (8)	
C1	0.2186 (8)	0.5785 (10)	0.5378 (4)	0.104 (2)	
N2	0.1332 (3)	0.0777 (4)	0.3021 (2)	0.0477 (9)	
H2A	0.0965	−0.0235	0.2886	0.057*	
C2	0.1419 (7)	0.4819 (12)	0.5800 (4)	0.095 (2)	
H2B	0.1174	0.5252	0.6264	0.114*	
C3	0.0997 (6)	0.3176 (11)	0.5537 (3)	0.0873 (18)	
H3A	0.0426	0.2492	0.5816	0.105*	
C4	0.1399 (5)	0.2505 (7)	0.4868 (3)	0.0650 (13)	
H4A	0.1112	0.1361	0.4704	0.078*	
C5	0.2215 (4)	0.3492 (6)	0.4436 (2)	0.0488 (11)	
C6	0.2666 (6)	0.5199 (7)	0.4691 (3)	0.0790 (16)	
H6A	0.3245	0.5898	0.4424	0.095*	
C7	0.2639 (4)	0.2853 (5)	0.3683 (2)	0.0421 (10)	
H7A	0.3576	0.3073	0.3647	0.051*	
C8	0.1895 (4)	0.3791 (5)	0.3012 (2)	0.0452 (10)	
H8A	0.1435	0.4870	0.3174	0.054*	
H8B	0.2487	0.4137	0.2626	0.054*	
C9	0.0967 (4)	0.2358 (5)	0.2735 (2)	0.0433 (10)	
C10	0.3413 (3)	−0.0333 (5)	0.3443 (2)	0.0385 (9)	
C11	0.4505 (4)	−0.0252 (6)	0.3943 (2)	0.0509 (11)	
H11A	0.4529	0.0611	0.4327	0.061*	
C12	0.5555 (4)	−0.1441 (7)	0.3875 (3)	0.0592 (13)	
H12A	0.6282	−0.1359	0.4203	0.071*	
C13	0.5499 (5)	−0.2750 (6)	0.3310 (3)	0.0660 (14)	
H13A	0.6185	−0.3569	0.3265	0.079*	
C14	0.4453 (4)	−0.2840 (6)	0.2825 (3)	0.0573 (12)	
H14A	0.4430	−0.3718	0.2447	0.069*	
C15	0.3405 (4)	−0.1633 (5)	0.2880 (2)	0.0423 (10)	
H15A	0.2699	−0.1706	0.2537	0.051*	

### Atomic displacement parameters (Å^2^)

**Table:**

	*U*^11^	*U*^22^	*U*^33^	*U*^12^	*U*^13^	*U*^23^
O	0.0586 (18)	0.0318 (16)	0.079 (2)	0.0078 (14)	−0.0066 (17)	0.0028 (15)
F	0.098	0.098	0.098	0.000	0.004	0.000
N1	0.0375 (18)	0.0253 (17)	0.059 (2)	0.0030 (14)	−0.0036 (16)	−0.0029 (15)
C1	0.125 (6)	0.083 (5)	0.101 (5)	0.018 (4)	−0.022 (5)	−0.058 (4)
N2	0.0402 (19)	0.0242 (17)	0.078 (3)	0.0014 (15)	−0.0117 (18)	0.0065 (16)
C2	0.089 (5)	0.132 (7)	0.063 (4)	0.044 (5)	0.007 (3)	−0.022 (4)
C3	0.081 (4)	0.117 (6)	0.064 (4)	0.015 (4)	0.002 (3)	0.008 (4)
C4	0.055 (3)	0.067 (3)	0.072 (3)	0.008 (3)	−0.002 (3)	0.000 (3)
C5	0.043 (2)	0.045 (3)	0.058 (3)	0.010 (2)	−0.004 (2)	−0.005 (2)
C6	0.109 (4)	0.063 (3)	0.065 (3)	−0.010 (3)	0.006 (3)	−0.027 (3)
C7	0.040 (2)	0.0212 (19)	0.066 (3)	−0.0020 (17)	0.0037 (19)	−0.0048 (19)
C8	0.040 (2)	0.030 (2)	0.066 (3)	−0.0010 (18)	0.007 (2)	−0.003 (2)
C9	0.042 (2)	0.031 (2)	0.057 (2)	0.0024 (19)	0.005 (2)	−0.013 (2)
C10	0.032 (2)	0.031 (2)	0.052 (2)	0.0004 (17)	−0.0015 (18)	0.0085 (19)
C11	0.047 (2)	0.049 (3)	0.056 (3)	0.014 (2)	−0.004 (2)	−0.009 (2)
C12	0.051 (3)	0.063 (3)	0.062 (3)	0.006 (2)	−0.017 (2)	0.009 (3)
C13	0.052 (3)	0.043 (3)	0.105 (4)	0.012 (2)	0.023 (3)	0.001 (3)
C14	0.055 (3)	0.042 (3)	0.076 (3)	0.006 (2)	0.024 (2)	−0.017 (2)
C15	0.038 (2)	0.039 (2)	0.050 (2)	−0.0045 (19)	0.0006 (18)	−0.0047 (19)

### Geometric parameters (Å, °)

**Table:**

O—C9	1.226 (5)	C6—H6A	0.9300
F—C1	1.398 (7)	C7—C8	1.552 (6)
N1—N2	1.404 (4)	C7—H7A	0.9800
N1—C10	1.408 (5)	C8—C9	1.487 (5)
N1—C7	1.500 (4)	C8—H8A	0.9700
C1—C2	1.317 (9)	C8—H8B	0.9700
C1—C6	1.405 (9)	C10—C15	1.381 (5)
N2—C9	1.311 (5)	C10—C11	1.404 (5)
N2—H2A	0.8600	C11—C12	1.394 (6)
C2—C3	1.354 (9)	C11—H11A	0.9300
C2—H2B	0.9300	C12—C13	1.389 (6)
C3—C4	1.370 (8)	C12—H12A	0.9300
C3—H3A	0.9300	C13—C14	1.353 (6)
C4—C5	1.366 (7)	C13—H13A	0.9300
C4—H4A	0.9300	C14—C15	1.398 (6)
C5—C6	1.400 (6)	C14—H14A	0.9300
C5—C7	1.502 (6)	C15—H15A	0.9300
			
N2—N1—C10	115.5 (3)	C8—C7—H7A	109.2
N2—N1—C7	105.8 (3)	C9—C8—C7	103.4 (3)
C10—N1—C7	118.9 (3)	C9—C8—H8A	111.1
C2—C1—F	120.9 (7)	C7—C8—H8A	111.1
C2—C1—C6	125.0 (6)	C9—C8—H8B	111.1
F—C1—C6	113.9 (7)	C7—C8—H8B	111.1
C9—N2—N1	115.1 (3)	H8A—C8—H8B	109.0
C9—N2—H2A	122.4	O—C9—N2	124.1 (4)
N1—N2—H2A	122.4	O—C9—C8	127.0 (4)
C1—C2—C3	117.9 (6)	N2—C9—C8	108.9 (3)
C1—C2—H2B	121.0	C15—C10—C11	118.0 (4)
C3—C2—H2B	121.0	C15—C10—N1	123.6 (3)
C2—C3—C4	121.1 (6)	C11—C10—N1	118.3 (3)
C2—C3—H3A	119.5	C12—C11—C10	121.2 (4)
C4—C3—H3A	119.5	C12—C11—H11A	119.4
C5—C4—C3	121.0 (5)	C10—C11—H11A	119.4
C5—C4—H4A	119.5	C13—C12—C11	119.0 (4)
C3—C4—H4A	119.5	C13—C12—H12A	120.5
C4—C5—C6	119.4 (4)	C11—C12—H12A	120.5
C4—C5—C7	123.0 (4)	C14—C13—C12	120.3 (4)
C6—C5—C7	117.6 (4)	C14—C13—H13A	119.8
C5—C6—C1	115.6 (6)	C12—C13—H13A	119.8
C5—C6—H6A	122.2	C13—C14—C15	121.1 (4)
C1—C6—H6A	122.2	C13—C14—H14A	119.4
N1—C7—C5	111.8 (3)	C15—C14—H14A	119.4
N1—C7—C8	103.6 (3)	C10—C15—C14	120.3 (4)
C5—C7—C8	113.6 (3)	C10—C15—H15A	119.8
N1—C7—H7A	109.2	C14—C15—H15A	119.8
C5—C7—H7A	109.2		
			
C10—N1—N2—C9	−128.7 (4)	C6—C5—C7—C8	−76.9 (5)
C7—N1—N2—C9	5.0 (5)	N1—C7—C8—C9	17.4 (4)
F—C1—C2—C3	−178.1 (5)	C5—C7—C8—C9	−104.2 (4)
C6—C1—C2—C3	−3.9 (11)	N1—N2—C9—O	−174.3 (4)
C1—C2—C3—C4	2.6 (9)	N1—N2—C9—C8	7.0 (5)
C2—C3—C4—C5	−1.4 (8)	C7—C8—C9—O	166.0 (4)
C3—C4—C5—C6	1.2 (7)	C7—C8—C9—N2	−15.3 (4)
C3—C4—C5—C7	−177.3 (4)	N2—N1—C10—C15	−6.1 (5)
C4—C5—C6—C1	−2.1 (7)	C7—N1—C10—C15	−133.5 (4)
C7—C5—C6—C1	176.5 (5)	N2—N1—C10—C11	176.8 (3)
C2—C1—C6—C5	3.6 (10)	C7—N1—C10—C11	49.4 (5)
F—C1—C6—C5	178.2 (5)	C15—C10—C11—C12	0.2 (6)
N2—N1—C7—C5	108.7 (3)	N1—C10—C11—C12	177.4 (4)
C10—N1—C7—C5	−119.4 (4)	C10—C11—C12—C13	−1.3 (7)
N2—N1—C7—C8	−13.9 (4)	C11—C12—C13—C14	1.4 (7)
C10—N1—C7—C8	117.9 (4)	C12—C13—C14—C15	−0.4 (7)
C4—C5—C7—N1	−15.1 (5)	C11—C10—C15—C14	0.8 (6)
C6—C5—C7—N1	166.3 (4)	N1—C10—C15—C14	−176.2 (4)
C4—C5—C7—C8	101.7 (4)	C13—C14—C15—C10	−0.8 (7)

### Hydrogen-bond geometry (Å, °)

**Table:**

*D*—H···*A*	*D*—H	H···*A*	*D*···*A*	*D*—H···*A*
N2—H2A···O^i^	0.86	1.92	2.777 (4)	172.
C4—H4A···N1	0.93	2.48	2.836 (6)	103.
C8—H8A···O^ii^	0.97	2.59	3.422 (5)	143.
C6—H6A···Cg3^iii^	0.93	2.96	3.866 (3)	165.
C12—H12A···Cg2^iv^	0.93	3.05	3.751 (3)	134.
C14—H14A···Cg3^v^	0.93	2.80	3.654 (3)	153.

Symmetry codes: (i) −*x*, *y*−1/2, −*z*+1/2; (ii) −*x*, *y*+1/2, −*z*+1/2; (iii) *x*, *y*+1, *z*; (iv) −*x*+1, −*y*, −*z*+1; (v) −*x*+1, *y*−1/2, −*z*+1/2.
